# Progress in investigating pituitary stalk lesions: A review

**DOI:** 10.1097/MD.0000000000041232

**Published:** 2025-01-10

**Authors:** Zaidong Zhang, Jinlin Wang, Yaru Shi, Yahui Zhao, Yanli Hu, Wentao Wang, Zonglan Chen

**Affiliations:** a Department of Hepatobiliary Surgery, Affiliated Hospital of Jining Medical University, Jining, Shandong, P.R. China; b Department of Clinical Medicine, Jining Medical University, Jining, Shandong, P.R. China; c Department of Emergency Medicine, Linyi People’s Hospital, Linyi, Shandong, P.R. China; d Department of Geriatrics, Taian Central Hospital, Taian, Shandong, P.R. China; e Department of Endocrinology and Metabolism, Affiliated Hospital of Jining Medical University, Jining, Shandong, P.R. China.

**Keywords:** hypopituitarism, pituitary stalk interruption syndrome, pituitary stalk lesions, pituitary stalk thickening, review

## Abstract

Pituitary stalk lesions are uncommon and are typically identified through pituitary magnetic resonance imaging and screening for causes of diabetes insipidus. Recent literature indicates that pituitary stalk lesions primarily manifest as pituitary stalk interruption syndrome and thickening of the pituitary stalk. The etiology of these lesions is complex and can be divided into major categories: congenital disorders, inflammatory or infectious diseases, and tumors. Therefore, achieving accurate diagnosis, differential diagnosis, and treatment for pituitary stalk lesions is crucial. This article aims to classify pituitary stalk lesions and delve into the latest research on their etiology, pathological mechanisms, clinical manifestations, diagnosis, and treatment of associated diseases.

## 1. Introduction

The pituitary stalk, or funnel or funiculus, is a funnel-shaped structure traversing the dura mater of the saddle diaphragm, establishing a connection between the hypothalamus and the pituitary gland.^[1,2]^ It exhibits a conical shape in its normal state, featuring a smooth surface with a broad upper part and a slender lower part. At the optic crossings, the transverse diameter was approximately 3.25 (SD, 0.56) mm, while at the pituitary insertion, the transverse diameter was approximately 1.91 (SD, 0.40) mm.^[3–5]^ The pituitary stalk can be categorized into an anterior nodal section (part of the anterior pituitary/adenohypophysis) housing a small number of gonadotrophic and corticotrophic cells and a posterior funnel section (part of the posterior pituitary/neurohypophysis) accommodating the unmyelinated axons of the large cellular supraoptic and para septal neurons. These axons terminate at the posterior pituitary gland, where they release oxytocin and arginine vasopressin into the bloodstream. Furthermore, the pituitary stalk is encircled by a convoluted ring of capillaries known as the Gomitoli, a component of the hypothalamic–pituitary portal system. Its primary role is to convey hormones and neurotransmitters released by the hypothalamus, thus regulating the functions of both the adenohypophysis and neurohypophysis.^[2]^

Pituitary stalk lesions are typically identified through pituitary magnetic resonance (MR) examination and etiologic screening for diabetes insipidus (DI). A large number of these patients are also detected while being investigated for pituitary hormone deficiency.^[3,5]^ However, there are a limited number of reports on pituitary stalk lesions in the literature, predominantly case reports. Consequently, the diagnosis, differential diagnosis, and treatment of pituitary stalk lesions are highly important in clinical practice. This paper comprehensively reviews the classification of pituitary stalk lesions; defines associated diseases; explores the etiology, pathology, clinical manifestations, diagnosis, and treatment; and provides an overview of related research progress.

## 2. Categorization of pituitary stalk lesions

**Figure 1.**
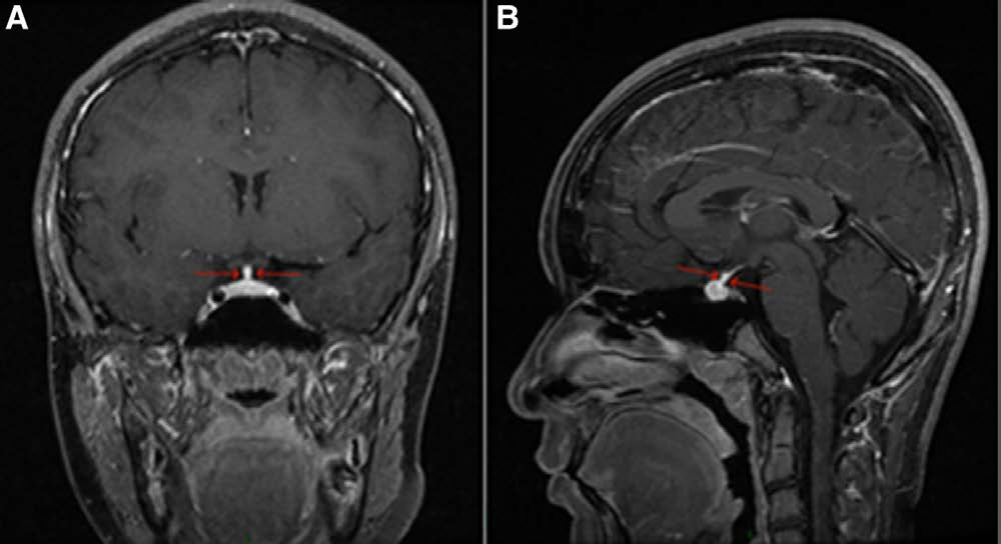
Thickening of the pituitary stalk: MRI direct dynamic enhancement scan of the pituitary gland (A: coronal, B: sagittal): the pituitary gland has an elevated diameter of approximately 6 mm (sagittal median level) and shows obvious uniform enhancement after enhancement; the pituitary stalk is centered, and the transverse diameter is thickened by approximately 3.3 mm. MRI = magnetic resonance imaging.

**Figure 2.**
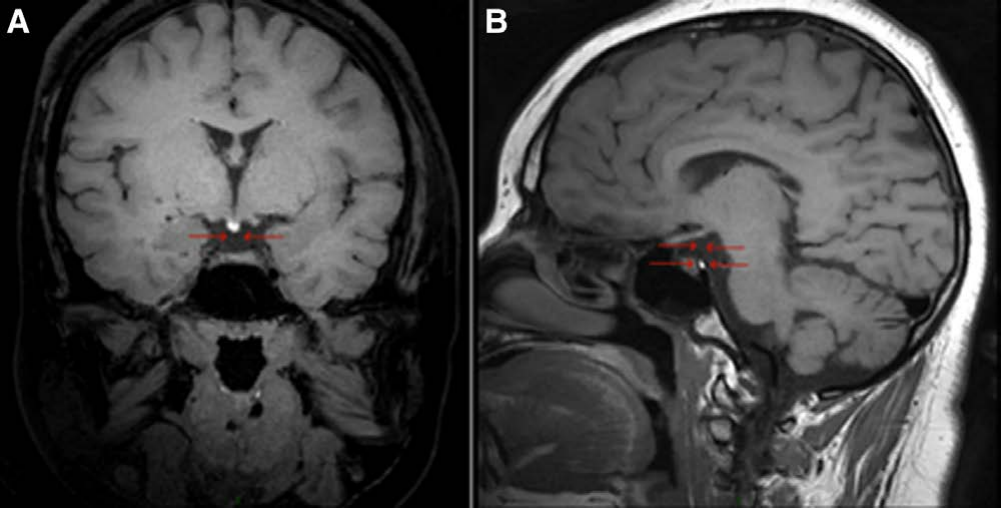
Pituitary stalk interruption syndrome/pituitary stalk agenesis/pituitary posterior lobe ectopic: MRI scan of the pituitary gland (A: coronal, B: sagittal): The pituitary volume is diminished, with a pituitary height diameter measuring approximately 3 mm. The pituitary stalk is absent, and there is ectopia of the posterior lobe of the pituitary. MRI = magnetic resonance imaging.

**Figure 3.**
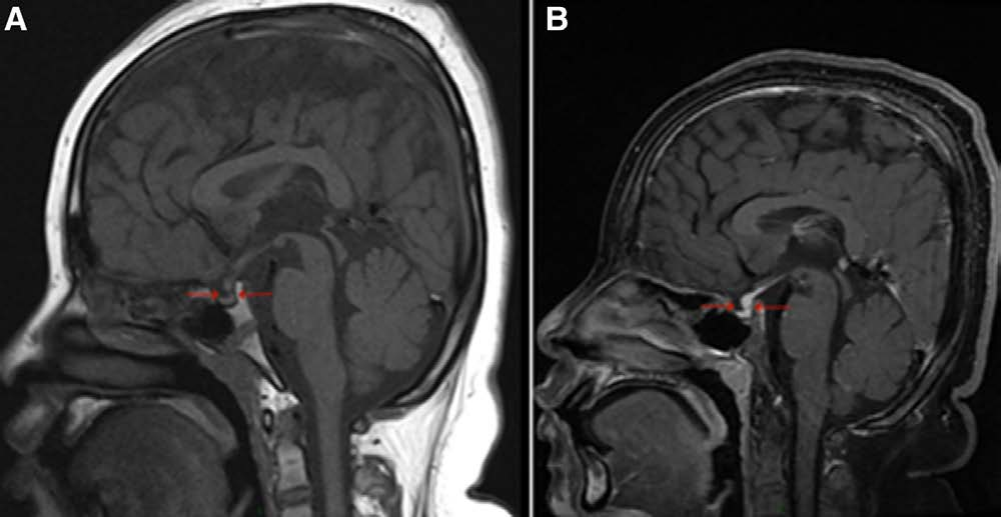
Pituitary hypoplasia (anterior pituitary dysplasia): (A) MRI scan of the pituitary gland: the pituitary stalk exhibits an enlarged diameter of approximately 4.2 mm, and there is an absence of a high signal in the neuropituitary gland on T1WI. (B) MRI-enhanced scan: the pituitary stalk appears slender in the lower part and thickened in the upper part, forming a nodular structure with a diameter of approximately 4.2 mm. MRI = magnetic resonance imaging.

**Figure 4.**
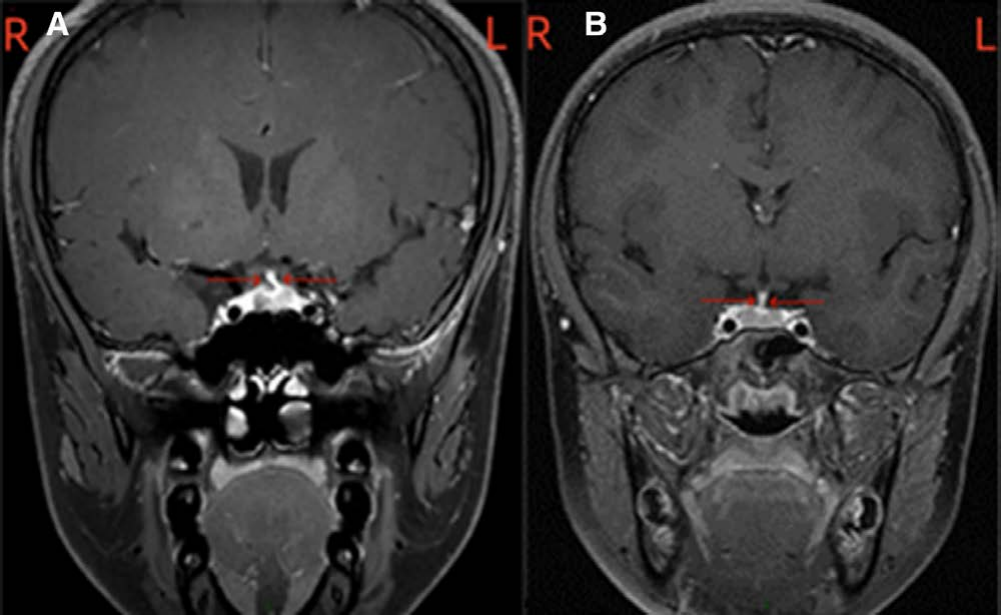
Pituitary stalk tilting: MRI direct dynamic enhancement scan of the pituitary gland: (A) the pituitary gland has a diameter of approximately 7.3 mm, and the pituitary stalk deviates to the left. (B) The median height diameter of the pituitary gland was approximately 6 mm, and the pituitary stalk was slightly to the right. MRI = magnetic resonance imaging.

**Figure 5.**
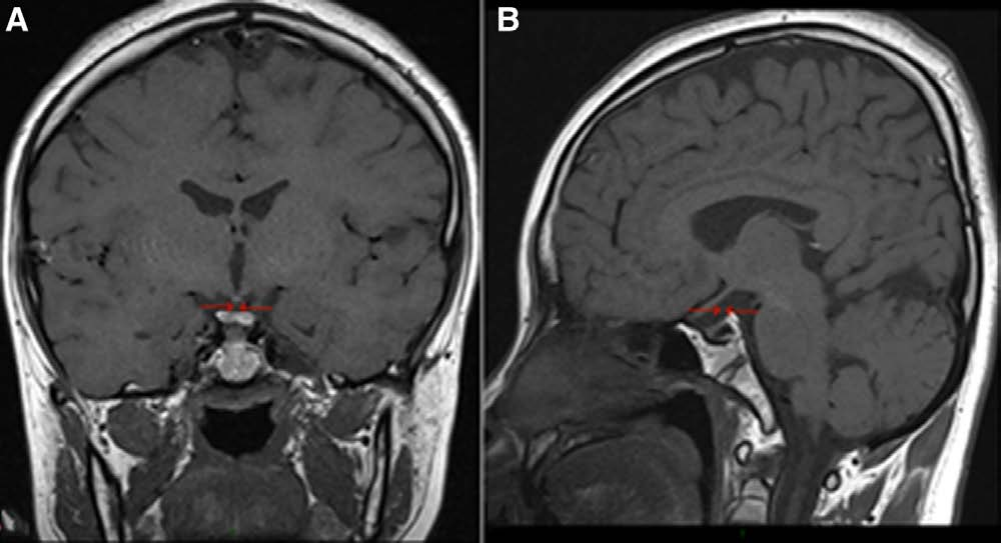
Pituitary stalk thinning: MRI scan of the pituitary gland (A: coronal, B: sagittal): the pituitary gland measures approximately 4 mm in height, with a thinned and poorly defined pituitary stalk. The high-signal neuropil is located superiorly to the dorsal aspect of the saddle. MRI = magnetic resonance imaging.

Pituitary stalk lesions were categorized based on etiology, with the results presented in Table 1. Additionally, classification by morphology is illustrated in Figures 1–5.

**Table 1:** Etiological classification of pituitary stalk lesions.

Etiology	Diseases	Morphological
Congenital	Pituitary hypoplasia	Agenesis, shortening, thickening, ectopic posterior pituitary, anterior pituitary dysplasia
	Pituitary stalk duplication	Pituitary stalk duplication
	Pituitary stalk interruption syndrome	Thin, agenesis, ectopic posterior pituitary, anterior pituitary dysplasia
	Rathke cleft cyst	Thickening
	Septooptic dysplasia	ectopic posterior pituitary, anterior pituitary dysplasia
Inflammatory	Hypophysitis (LYH)	Thickening
	Hypophysitis (IgG4)	Thickening
	Sarcoidosis	Thickening
	Tuberculosis	Thickening
	Pituitary abscess	Thickening
	Histiocytosis: Langerhans cell histiocytosis (LCH)	Thickening
	Histiocytosis: Erdheim-Chester Disease	Thickening
	Histiocytosis: Xanthoma disseminatum	Thickening
	Wegener granulomstosis	Thickening
	Whipple disease	Thickening
	Parasitism	Thickening
*Neoplastic*		
Local	Germinal cell tumors	Thickening
	glioma	Thickening
	Craniopharyngioma	Thickening
	Pituitary adenoma	Thickening
	Meningioma	Thickening
	Pituicytoma	Thickening
	Lymphoma primary in CNS	Thickening
	Leukemia/Lymphoma systemic	Thickening
Metastases	Lung cancer	Thickening
	Breast cancer	Thickening
	Thyroid cancer	Thickening
	Stomach cancer	Thickening
	Skin cancer	Thickening
Other	Cavernous hemangioma	Thickening
	Choroid plexus carcinoma	Thickening
	Schwannoma	Thickening
	Spindle cell carcinoma	Thickening

*Note*: Definition of pituitary stalk thickening: ≥3 mm in diameter.

CNS = central nervous system, LYH = lymphocytic pituitary hypophysitis.

## 3. Etiologies

### 3.1. Inflammatory

#### 3.1.1. Histiocytosis

Langerhans cell histiocytosis (LCH) is a relatively rare disease that affects multiple organs and systems, including the skin (30%), bones (80%), and hypothalamo-pituitary gland (25%).^[6]^ It is characterized by abnormal clonal proliferation and aggregation of epidermal dendritic cells.^[5,7–9]^ The classification of LCH is controversial, as some scholars categorize it as a congenital disease, while others classify it as an inflammatory or neoplastic disease.^[5,7,9–11]^ Several studies have reported the presence of the BRAF V600E mutation in LCH patients. It is hypothesized that this mutation may be linked to the development of LCH and could serve as a key biomarker in the future. However, the specific etiology of LCH remains controversial.^[12–15]^

Patients with LCH are usually asymptomatic or present with mild symptoms such as fatigue, generalized weakness, weight loss, night sweats, nausea, itching, and fever.^[7]^ Neurological involvement frequently results in central diabetes insipidus (CDI) and hypopituitarism, characterized by deficiencies in growth hormone(GH), gonadotropins, adrenocorticotropic hormone (ACTH), and thyroid-stimulating hormone (TSH), as well as hyperprolactinemia.^[5,8,9,16,17]^ The involvement of other systems is evident through corresponding symptoms: the skeletal system may cause pain,^[2]^ and there may be skin lesions such as seborrheic dermatitis, gingival hypertrophy, oral ulcers, enlarged lymph nodes, and lung involvement.^[7,9]^

The diagnosis of LCH involving the pituitary gland/pituitary stalk is established through MR, and typical manifestations include diffuse thickening of the pituitary stalk with isosignal on T1WI and hypersignal on T2-weighted images (T2WI), along with the absence of enhancement in the posterior pituitary lobe.^[5,8,10,18–21]^ Niu et al compared the diagnostic efficacy of 18F-FDG PET (positron emission tomography) with magnetic resonance imaging (MRI) for early staging and follow-up of LCH. They discovered that 18F-FDG PET was more precise at evaluating disease activity after chemotherapy, while MRI exhibited superior overall sensitivity compared to PET.^[22]^ Recent studies indicate that 18F-FDG PET/MR offers improved detection and distribution of lesions across different organ systems than traditional methods, though it lacks the anatomical sensitivity of MRI,^[21]^ which indicates that a combination of MRI/PET may be employed in the future to enhance diagnostic accuracy. Importantly, MRI findings in LCH patients lack specificity. Conditions such as lymphocytic pituitary inflammation, germ cell tumors (GCTs), and tuberculosis can present with similar features. Consequently, an accurate diagnosis still relies on histopathological biopsy. Positive immunohistochemical staining for CD1a, S100, and/or CD207 can play a crucial role in guiding pathological biopsies.^[8,23,24]^

The diagnosis of LCH is inherently complex, potentially attributed to the heterogeneity of inflammatory changes and variations in lesion depth, as suggested by Oda et al.^[8]^ The primary challenge in differentiating between GCTs and lymphocytic leaking pituitary inflammation lies. Distinguishing these tumors from GCTs is relatively simple: cerebrospinal fluid markers such as PLAP, human chorionic gonadotropin, and alpha-fetoprotein can be detected in the serum and cerebrospinal fluid. However, distinguishing pulmonary inflammation from lymphocytic leaking pituitary inflammation typically necessitates histopathological biopsy.^[8,25,26]^

There is no established optimal treatment for LCH. Possible treatment approaches include mere observation, surgical intervention, low-dose radiotherapy, and chemotherapy.^[27]^ Shinsaku Imashuku et al from the LCH Study Group in Japan, proposed a pharmacological regimen comprising vincristine, methotrexate, and steroids.^[8,28]^ Spontaneous regression of the disease has been reported in several cases involving multiple systems, including the skin, bones, lungs, and pituitary stalks.^[9,29]^

Erdheim-Chester disease is a rare non-Langerhans histiocytosis that frequently affects the hypothalamic-pituitary region, shares clinical features with LCH, and often impacts multiple organ systems. Nervous system involvement can result in CDI, headache, visual disturbances, cranial nerve palsies, and hypopituitary symptoms.^[30]^ Skeletal disorders often involve symmetrical diaphysis and epiphysis, and renal and cardiovascular pathologies can also occur.^[31]^ Yi Tang et al conducted a retrospective analysis of 48 patients diagnosed with Erdheim-Chester disease at Peking Union Medical College Hospital. The study revealed that CDI was the most prevalent symptom in patients with pituitary involvement. MRI findings indicated a loss of signal in the posterior lobe of the pituitary gland on T1WI and thickening of the pituitary stalk.^[32]^

Xanthoma disseminatum is a rare non-Langerhans cell histiocytosis characterized by widespread xanthomatous lesions affecting the skin.^[11,33]^ Shuaihantian Luo et al reported the first case of a pediatric patient with DI and a BRAF mutation. The main clinical manifestations included generalized dermatological lesions and DI. MRI revealed an enlarged pituitary gland and thickened pituitary stalk.^[33]^ Cone-like enhancement on MRI of disseminated xanthomas involving the pituitary stalk has also been reported.^[11]^ Kobayashi et al reported a case of disseminated xanthoderma arising from a panhistiocytic histiocytoma. The patient exhibited clinical manifestations of a xanthoderma-like rash and CDI. MRI revealed thickening of the pituitary stalk and high signal loss in the posterior lobe of the pituitary gland, consistent with the findings of Shuaihantian Luo et al. Pathological examination of xanthoderma papillarum tissues showed infiltration of dermal tissues by foamy and giant cells.^[34]^ In Tae-Kyu Lee et al.’s report, a patient who developed disseminated juvenile yellow granuloma following LCH treatment exhibited thickening and enhancement of the pituitary stalk on MRI similarly.^[35]^

#### 3.1.2. Hypophysitis

Primary hypophysitis can be categorized into various types based on histological and radiological criteria. Histologically, it includes 2 main forms: lymphocytic and granulomatous (sarcoidosis), both of which fall under the umbrella of autoimmune hypophysitis. Lymphocytic hypophysitis (LYH), the more prevalent type, is an autoimmune condition affecting the pituitary gland.^[36,37]^ When inflammation is limited to the funiculus and posterior lobe, it is termed funicular- pituitary inflammation according to the radiological anatomical site classification.^[5,7,37,38]^ Lymphocytic funnel neurohypophysitis (LINH) was initially described in 1970 by Sito et al. It is recognized as one of the leading causes of inflammation in pituitary stalk abnormalities.^[39,40]^

Primary pituitary inflammation commonly presents with symptoms such as headache, visual impairment from optic nerve compression, hypopituitarism, and hyperprolactinemia.^[37,41]^ LYH is characterized by 4 primary symptoms: saddle area compression, hypopituitarism, CDI, and hyperprolactinemia. Among these, secondary adrenal insufficiency and secondary hypothyroidism are the most frequent endocrine disorders associated with LYH.^[9]^ In a study by Doknic et al., 2 patients with pituitary inflammation had DI, while all 6 had partial hypopituitarism, primarily in the form of hypogonadotropic hypogonadism and growth hormone deficiency.^[42]^ In a retrospective study of 50 patients with LYH, Wang Shuchang et al reported that CDI, which accounts for up to 72.0% of patients, was the most common endocrine dysfunction. In contrast, anterior pituitary dysfunction and adrenocortical hypofunction were less common at presentation. These findings are consistent with those of previous studies.^[43]^ Saddle compression symptoms are infrequent in LINH patients. Notably, inflammation in LINH is often self-limiting, while DI typically presents as irreversible.^[7,9]^

While MRI is the preferred method for definitively diagnosing primary pituitary inflammation, biopsy remains the gold standard for LYH diagnosis.^[37]^ Imaging features include enlarged triangular or dumbbell-shaped glands with thickened pituitary stalks that are not markedly oblique.^[38,44]^ Pathologically, LYH is characterized by a lymphocytic infiltrate with a predominance of T cells, especially CD4 cells, along with plasma cells, eosinophils, macrophages, histiocytes, and neutrophils.^[9]^ However, due to the risk associated with pituitary biopsy, this procedure is not recommended except for special cases, such as when malignancy needs to be excluded.^[7,9]^ According to several scholars, pituitary stalk thickening is considered the most robust predictor of inflammatory processes in the pituitary gland.^[38,44]^ Literature reports have consistently demonstrated a distinct MRI presentation of lymphocytic leaking neuropituitarism (LINH): diffuse thickening of the pituitary stalk.^[5,7,9,10,39,40,42]^ MRI features of LYH include pituitary stalk thickening, diffuse pituitary gland enlargement, abnormal signals in the posterior pituitary lobe, and focal invasion of the cavernous sinus and adjacent areas.^[10]^ Gutenberg et al demonstrated that autoimmune pituitary hyperplasia can be distinguished from nonsecretory pituitary adenomas through radiological features, including a symmetric enlargement of the pituitary gland, a thickened nontapering pituitary stalk, an intact sellar floor and the absence of a high signal in the posterior pituitary lobe. Conversely, pituitary macroadenomas typically exhibit asymmetrical growth, often displace the funiculus, and rarely involve the stalk or erode the sellar floor.^[36]^ In a retrospective study, Wang Shuchang et al investigated 50 patients with LYH at Peking Union Medical College Hospital from 1999 to 2016 and revealed that the most prevalent imaging feature was pituitary stalk thickening (96.0%).^[43]^ Symptoms and neurological imaging findings in LCH patients often resemble those in LINH patients, making it challenging to distinguish them solely through MRI; consequently, additional biopsy is frequently necessary for accurate identification.^[8]^ Rabphilin-3A is a highly reliable blood biomarker in LYH patients and has a high sensitivity for detecting LYH. Moreover, this new biomarker can assist in distinguishing between LINH and LCH.^[8,37,45]^

Treatment for pituitary inflammation relies mainly on symptomatic treatment, involving approaches such as mass reduction, anti-inflammatory therapy, and hormone replacement therapy, while surgery or radiotherapy is less commonly employed.^[9,38,41,43]^ High-dose glucocorticoids are the preferred treatment for symptoms such as headaches and reduced vision resulting from mass effect in sella and compression of surrounding structures. For patients resistant to steroids or unable to tolerate their side effects, incorporating additional immunosuppressive medications may be advisable.^[41,46]^ Surgery is recommended only when the disease is severe, not responsive to pharmacological treatment, or has caused significant deficiencies. Radiotherapy may be considered for patients who do not respond to pharmacological treatment, who experience recurrent disease, or who exhibit severe mass effect symptoms.^[9,37,46,47]^

Numerous studies have revealed that IgG4-related pituitary inflammation can affect the anterior and posterior lobes of the pituitary gland as well as the pituitary stalk, primarily manifesting as pituitary stalk thickening. This condition typically presents with urolithiasis, indications of pituitary hypoplasia, or symptoms associated with mass effects in the saddle region, such as headaches or visual impairments.^[37,48]^ Imaging for IgG4-related pituitary diseases often reveals stalk thickening. For instance, Alireza et al conducted a retrospective analysis of 115 patients with IgG4-related pituitary inflammation, finding that 90 patients exhibited stalk thickening on MRI. The most effective treatment for IgG4-related pituitary inflammation remains uncertain.^[48]^ Sbardella et al described 3 patients experiencing pituitary inflammation due to ibritumomab. Despite not undergoing biopsy, these patients showed marked improvement in symptoms, clinical signs, and imaging results following the cessation of the drug, without necessitating any specific treatment.^[3]^

#### 3.1.3. Sarcoidosis

Sarcoidosis is an autoinflammatory granulomatous disease of unknown origin that typically affects multiple systems. The most common manifestations include enlarged hilar lymph nodes and interstitial lung infiltrates.^[7,9]^ While the neurological impact of sarcoidosis is relatively rare, affecting approximately 5% of patients, it can involve various regions, including the brain, spinal cord, meninges, cranial nerves, and peripheral nerves.^[7,49]^

The clinical manifestations of sarcoidosis include fatigue, weight loss, fever, persistent cough, skin changes (such as papules, nodules, subcutaneous infiltrates, erythema nodosum, and lupus), ocular lesions (uveitis, retinal changes, conjunctival nodules, and loss of vision), and enlargement of peripheral lymph nodes.^[7,10]^ The accumulation of sarcoidosis in the hypothalamus and pituitary regions may result in symptoms, including DI, hyperprolactinemia, decreased testosterone, luteinizing hormone, and follicle-stimulating hormone (FSH) levels, as well as delayed growth and sexual development.^[5,9,10,50]^ Moszczyńska et al summarized a diverse range of symptoms associated with central nervous system (CNS) sarcoidosis, including seizures, headache, vomiting, lethargy, cranial neuropathy, and hypothalamic dysfunction.^[9]^ Elina et al recently reported a patient who exhibited progressive gait disturbance, cognitive dysfunction, hypothermia, bradycardia, and hypopituitarism, consistent with previous findings.^[51]^

Enhanced MRI is the preferred modality for studying CNS sarcoidosis.^[11]^ Nonetheless, according to Hána et al, MRI is considered to lack specificity for sarcoidosis due to its diverse presentation.^[7]^ In the majority of patients with CNS sarcoidosis, MRI might reveal an enhanced signal in the pituitary stalk, thickening of the pituitary stalk, or enlargement of the pituitary gland.^[5,10,11,51]^ In situations where diagnosis is challenging, chest radiographs and CT scans of the lungs are valuable for identifying lung-specific lesions and aiding in further diagnosis.^[2,5,7]^ Recent literature also suggests that 18F-FDG PET can be beneficial as an adjunctive tool for diagnosing CNS sarcoidosis.^[52–54]^ There is strong evidence that pathological biopsies confirm the diagnosis of this disease.^[7,9]^ Several studies have documented diverse laboratory manifestations associated with sarcoidosis, including elevated serum and cerebrospinal fluid angiotensin-converting enzyme levels,^[55,56]^ an increased CD4/CD8 lymphocyte ratio in alveolar lavage fluid,^[7]^ hypercalcemia and hypercalciuria,^[57]^ elevated nonspecific liver enzymes^[7]^ and cerebrospinal fluid composition abnormalities.^[55,58]^ These findings may be linked to sarcoidosis, although normal values do not exclude the diagnosis of sarcoidosis.^[7,9]^ However, further research is needed to establish specific correlations between these indices and sarcoidosis incidence. Diagnosing sarcoidosis is an exclusionary process involving a comprehensive assessment that includes history, physical examination, clinical presentation, laboratory tests, MRI, CT, chest radiographs, and biopsy.^[7,9]^ Special attention should be given to ruling out lymphocytic pituitary inflammation, as distinguishing between isolated CNS sarcoidosis and lymphocytic pituitary inflammation often requires pathological biopsy.^[59]^

Corticosteroids, especially glucocorticoids, have been demonstrated to be effective at treating sarcoidosis and have therapeutic efficacy. It is important to note that in patients with DI, managing polyuric symptoms may pose greater challenges.^[5,9,10]^ If glucocorticoid therapy proves ineffective, alternative second-line medications such as methotrexate, azathioprine, mycophenolate mofetil, and leflunomide or third-line options such as TNF-alpha antagonists such as infliximab and adalimumab may be considered for treatment.^[9,60–62]^ Daan Fritz et al conducted a study that revealed a 70% improvement in symptoms among patients treated with infliximab for neurotypical nodular disease.^[63]^ D. Sofia Villacis-Nunez and colleagues treated a 17-year-old female patient with neurotypical tuberculosis using corticosteroids, methotrexate, and adalimumab, leading to a gradual normalization of the pituitary lesion, as observed by imaging.^[64]^

#### 3.1.4. Pituitary tuberculosis

Tuberculosis involvement in the pituitary stalk or gland is highly uncommon, representing only 1% of lesions affecting the anterior and/or suprasellar regions.^[10,42,65,66]^ Tuberculosis can lead to the formation of tuberculous balls or granulomas in the pituitary stalk, while tuberculomas are infrequent and primarily observed in women.

Individuals affected by pituitary tuberculosis might exhibit hypopituitarism alongside the symptoms of primary systemic tuberculosis.^[5,10,42,67]^ Previous reports have shown that patients with pituitary tuberculosis present with secondary hypothyroidism and hyperprolactinemia,^[67]^ although hypopituitarism is uncommon in pituitary tuberculomas.^[66]^ Additional potential symptoms include headache, vomiting, visual disturbances, amenorrhea, galactorrhea, diplopia, generalized apathy, and weight gain.^[68,69]^

On MR images, pituitary tuberculosis might exhibit pituitary stalk thickening, enhanced adjacent meninges, and paranasal sinus or nasal fossa involvement. Tuberculomas appear isohypointense on T1WI and hyperintense on T2WI.^[5,10,68]^ MRI of a 54-year-old female patient revealed an enlarged pituitary gland with thickening of the pituitary stalk, showing a low signal on T1WI and a high signal on T2WI, consistent with previous findings.^[70]^ It is crucial to recognize that pituitary adenomas may exhibit similar imaging findings. Therefore, a comprehensive assessment is necessary, including a history of tuberculosis, systemic tuberculosis symptoms, and laboratory investigations (tuberculin test, blood sedimentation, etc).^[10]^

Treatment for pituitary tuberculosis involves antituberculosis therapy, including quadruple therapy (isoniazid, rifampicin, ethambutol, and pyrazinamide) and dual therapy (isoniazid, rifampicin). Hypopituitarism rarely occurs in pituitary tuberculosis tumors, leading to scarce literature on hormone replacement therapy. Furthermore, the use of glucocorticoids in treating pituitary tuberculosis is debated.^[66]^ The patient’s overall condition significantly improved with the reintroduction of antituberculosis therapy, hormone replacement therapy, and ventriculoperitoneal shunt surgery, as reported in the case of an 18-year-old female.^[69]^ Additionally, cases of pituitary stalk/funnel enhancement due to tuberculous meningitis have been documented, although these cases are infrequent.^[71]^

#### 3.1.5. Pituitary abscess

Pituitary abscess (PA) is a rare infectious disease affecting the pituitary gland that represents only 1% of all pituitary diseases and has a high prevalence in women.^[72,73]^ PAs typically manifest as an enlarged mass in or around the saddle region, potentially affecting nearby anatomical structures. This can include thickening of adjacent meninges, thickening of the pituitary stalk, unilateral or bilateral encroachment on the cavernous sinus, compression leading to displacement of the optic nerve crossings and the third ventricle, and potential involvement of the mucosa of the paranasal sinuses.^[72]^ In the study by Zhi et al, all 4 patients with PA demonstrated thickening of the pituitary stalk.^[73]^ In the study by Zhi et al, all 4 patients with PA demonstrated thickening of the pituitary stalk. PA is categorized as primary or secondary based on etiology, with primary PAs being the most prevalent. Primary PA is primarily caused by gram-positive and gram-negative bacteria, and fungi are infrequently implicated. In contrast, secondary PAs are more commonly associated with fungal infections.^[74]^

The clinical manifestations of PA lack specificity and may include headache, dizziness, vomiting, fever, visual disturbances, lethargy, behavioral abnormalities, anterior pituitary hypopituitarism (e.g., anorexia, amenorrhea), and posterior pituitary hypopituitarism (DI, irritable thirst).^[72,73,75–78]^ Zhi et al’s study reported 4 patients with PA involving the pituitary stalk. The main manifestations are headache, fever, weakness of the extremities, and sexual dysfunction.^[73]^

On MRI, PAs exhibit a combination of hypointense and isointense signals on T1WI and a combination of isointense and hyperintense signals on T2WI. Enhancement scans reveal saddle ring enhancement, which is considered a characteristic imaging manifestation of PAs.^[73,77]^ Mature abscesses typically exhibit classic rim enhancement, while immature abscesses often display solid enhancement with specific signs of atypical rim enhancement. In patients where inflammation extends to the pituitary stalk, MRI commonly reveals thickening and enhancement of the pituitary stalk.^[72]^ The thickened pituitary stalk has a greater diameter at the lower end than at the upper end, indicating that the inflammation spreads upward from the bottom.^[73]^ Diffusion-weighted imaging may be useful in differentiating PAs from other cystic lesions and necrosis or infarction.^[72,78]^

Contemporary approaches for treating PAs include surgical interventions via the nasopharyngeal sinus route, antibiotic therapy, and hormone replacement therapy. Numerous reports in the literature highlight the favorable prognosis observed in patients with PAs when treated with a combination of antibiotics and the application of endoscopic transsphenoidal surgery (TSS).^[72,73,75–78]^ In a study by Gao et al,^[74]^ 66 out of 6361 patients treated for pituitary disorders at Peking Union Medical College Hospital were diagnosed with PAs. Of these, 69.7% (46 patients) developed varying degrees of hypopituitarism post-surgery. The most common deficiency was in ACTH, affecting 48.5% (32 patients), treated with prednisone acetate. Hypothyroidism, observed in 10.6% (7 patients), was treated with levothyroxine. 6.1% (4 patients) exhibited both gonadotropin deficiency and hypothyroidism, treated with prednisone acetate and levothyroxine. The least common condition, hypogonadotropic hypogonadism, affected 4.5% (3 patients) and was managed with sex hormone replacement therapy. Four patients did not follow medical advice and discontinued hormone replacement therapy, leading to life-threatening pituitary crises. Christopher et al reported a rare case in which a patient developed complications of a PA after undergoing transnasal endoscopic drainage of a suprasellar arachnoid cyst.^[79]^ Therefore, TSS is likely to be a risk factor contributing to PA.^[78]^ This finding suggested that surgical safety should be accurately assessed when treating PAs to prevent disease deterioration. Antibiotic therapy primarily involves preoperative oral and perioperative intravenous administration. It is initiated orally 2 weeks after surgery and is continued for a minimum of 2 weeks.^[72]^

#### 3.1.6. Wegener granulomatosis

Wegener granulomatosis, also referred to as granulomatous polyangiitis (GPA), is a multisystemic autoimmune disease marked by necrotizing granulomatous inflammation of small blood vessels.^[80]^ The disease can impact various organs and systems, such as the lungs, kidneys, skin, and nervous system. However, the inclusion of the pituitary gland and pituitary stalks is exceedingly rare, constituting <1% of all GPA patients.^[80–82]^

GPA can induce multisystem symptoms involving the ears, nose, throat, eyes, meninges, lungs, and bones/joints.^[80,81,83]^ Common manifestations of the disease include CDI and reduced gonadotropin levels. The predominant symptoms of GPA-associated pituitary infections are polydipsia and polyuria. Other potential symptoms include headache, visual impairment, and a range of pituitary dysfunctions, including hypogonadotropic hypogonadism, lactotropic hormone regulation abnormalities, ADH deficiency, central hypothyroidism, hyperprolactinemia, central hypocortisolism, growth hormone deficiency, hypoprolactinaemia, and overall pituitary hypopituitarism.^[80–82]^ Concerning patients with GPA, Vega-Beyhart analyzed 197 cases reported from 1950 to 2019. Only 4 patients exhibited pituitary and pituitary stalk involvement (pituitary stalk thickening).^[80]^ These 4 patients manifested upper respiratory symptoms, spontaneous rhinorrhea, fever, hematuria, weight loss, fatigue, polydipsia, hearing loss, generalized facial paralysis, headache, and nasal congestion, indicative of multisystem involvement.

The primary MRI feature of pituitary inflammation associated with GPA includes a mass in the saddle region. The pituitary gland exhibits enlargement on T1WI and a reduced enhancement signal in the posterior pituitary lobe.^[80,81]^ In cases where the pituitary stalk is affected, both thickening of the stalk and enlargement of the pituitary funnel may be noted.^[80,84]^ Biopsy rates for GPA are low, with only 28% of patients undergoing diagnostic biopsy in the study by Vega-Beyhart et al.^[80]^ This low rate could be associated with c-ANCA positivity, which might enhance the accuracy of GPA diagnosis to some extent. However, in a retrospective study of 12 GPA patients conducted by Gu, Y. et al, nearly all were ANCA-negative when lung and kidney involvement was absent.^[81]^ This finding indicates that ANCAs may lack sufficient sensitivity for diagnosing GPA in patients with pituitary gland involvement, and a negative ANCA does not entirely rule out GPA diagnosis.

The primary treatment modalities for GPA involve pharmacological and surgical approaches. Pharmacological intervention is the cornerstone in managing GPA-associated pituitary inflammation, and commonly prescribed regimens comprising glucocorticoids combined with cyclophosphamide are used.^[80]^ Additional therapeutic options include biologics (e.g., rituximab and infliximab) and other immunosuppressive agents, such as methotrexate and azathioprine.^[82,83,85]^ For instance, azathioprine is frequently utilized for disease maintenance therapy, and a semiannual, low-dose rituximab maintenance infusion can decrease the risk of disease recurrence, given an acceptable safety profile. Long-term hormone replacement therapy may be necessary for patients with hormone deficiencies.^[86]^ Surgical intervention is mainly employed for patients in need of decompression surgery to alleviate the effects of the mass.^[80]^ Vega-Beyhart et al reported that 4 patients with GPA-associated pituitary stalk involvement experienced effective relief of symptoms through a combined regimen of hormones along with monoclonal antibodies or azathioprine.^[80]^ Matthew et al reported a case of recurrent GPA symptoms in a patient with a history of GPA for more than 20 years who had not undergone maintenance therapy. Cranial MRI revealed inflammation involving the pituitary gland and pituitary stalks, and the patient was successfully treated with immunosuppressive therapy, yielding positive outcomes.^[86]^ Ozlem Celik et al also reported a case in which a GPA patient experienced severe headaches and bilateral temporal hemianopsia after 10 years of glucocorticoid replacement therapy. These symptoms were alleviated through transnasal-TSS and rituximab, resulting in the relief of the patient’s headache and the restoration of vision.^[84]^

#### 3.1.7. Other rare diseases

Whipple disease, caused by gram-positive Trichosporon bacillus, is a chronic systemic condition.^[87]^ Although extremely rare, it can lead to pituitary stalk lesions. However, such cases have been poorly reported in the recent literature. Whipple disease has been associated with thickening of the pituitary stalk, as suggested by Rupp et al Individuals with a history of Whipple disease treated with tetracycline and presenting with pituitary stalk or hypothalamic lesions should be highly suspected of having recurrent CNS disorders related to Whipple disease.^[5,88]^

Pituitary stalk lesions caused by parasites are exceptionally rare. Among the parasitic diseases affecting the CNS, cerebral cysticercosis is one of the most common. Cysts in the saddle region are in proximity to the pituitary stalk and pituitary gland, increasing susceptibility to damage. This damage can result in symptoms such as headache and visual impairment. MRI may reveal funnel enhancement and thickening in affected patients. Unfortunately, cysticidal drugs are ineffective, and surgical removal remains the sole effective treatment for this disease.^[89]^

### 3.2. Neoplastic

#### 3.2.1. Germinal cell tumors

Primary CNS GCTs are tumors that originate from germinal cells during embryonic development and migrate to the CNS.^[7]^ These tumors predominantly occur in young individuals, typically before the age of 20.^[5,7,9,10]^ According to the 2021 WHO Classification of Tumors of the Central Nervous System,^[90]^ GCTs include various types, such as GCTs, mature teratomas, immature teratomas, teratomas with somatic-type malignancies, embryonal carcinomas, yolk sac tumors, choriocarcinomas, and mixed GCTs. Germ cell tumors typically manifest as masses in the hypothalamic or pineal regions, with a higher prevalence observed in the hypothalamic-pituitary area.^[5,7]^

When GCTs accumulate in the pituitary stalk, potential clinical manifestations include DI, generalized headache, nausea, severe back pain, vision changes, increased intracranial pressure or neurological symptoms, and pituitary hypoplasia, particularly affecting GH secretion and, to a lesser extent, TSH, luteinizing hormone, and FSH secretion.^[5,7,9,10,91]^ Several studies have highlighted that HCG-producing GCTs can present with GnRH-independent precocious puberty. Therefore, in instances of peripheral precocious puberty, it is crucial to assess cerebrospinal fluid β-hCG levels. If elevated, a brain MRI should be contemplated for further evaluation.^[92,93]^ CDI is typically regarded as the initial symptom of pituitary GCTs, and at times, CDI may manifest before radiological abnormalities in neuropituitary GCTs.^[9]^

MRI findings may include thickening of the pituitary stalk, which is the most common manifestation; loss of a high signal in the neuropituitary gland on T1WI, with the lesion being isosignal or mildly hypersignal compared to the normal pituitary gland; and decreased contrast uptake.^[9,10,19]^ MRI features are not specific, and several other conditions may present similarly. Therefore, it is essential to complement this with additional investigations. For instance, Natascia et al proposed that diffusion-weighted imaging is valuable for distinguishing suprasellar GCTs from craniopharyngiomas, gliomas, and other common childhood suprasellar tumors.^[22]^ Additionally, PET/CT may aid in differentiating GCTs from LCH.^[5]^ Many scholars suggest that the accumulation of a tumor in both the suprasellar and pineal glands, without further spread, strongly indicates a GCT.^[7,22,26,91,94]^ Pathological biopsies remain a robust diagnostic tool for this disease. Moreover, markers like human chorionic gonadotropin and alpha-fetoprotein, secreted by GCTs, aid in diagnosis, though their sensitivity is limited. In Allen et al’s study, 60% of patients with GCTs had normal serum and cerebrospinal fluid β-hCG levels, with only 5% showing abnormal values.^[95,96]^

Other tumor markers, such as β-hCG and placental alkaline phosphatase-GCT, have been linked to GCTs, but the exact correlation still requires additional study.^[9,97]^

Germ cell tumors exhibit high sensitivity to radiotherapy, and chemotherapy can be used as a supplement to reduce the radiotherapy dose. Simple GCTs are less aggressive than other GCTs and mixed types of tumors and exhibit a relatively better response to radiotherapy.^[5,7,10]^

#### 3.2.2. Glioma

Glioma, also referred to as glioblastoma, is the most prevalent primary intracranial tumor that arises from neuroepithelial cells and is highly malignant. These tumors can be categorized into astrocytoma, glioblastoma, and other types based on the morphology of the cell types. Marina et al summarized clinical studies on malignant gliomas originating in the hypothalamus/pituitary axis, specifically within the intrasaddle region. These studies revealed presentations marked by acute neurological symptoms (such as vomiting and headache), visual disturbances, endocrine impairments (including amenorrhea, breast milk spillage, and DI), and loss of appetite and fatigue.^[98]^ MRI revealed a high signal in T1-weighted images (T1WIs), a low signal or isosignal signal in T2WIs, and pituitary stalk thickening when affected. Nevertheless, analogous imaging features are observed in pituitary adenomas, glioblastomas, and squamous papillary craniopharyngiomas, complicating the differential diagnosis of gliomas.^[98]^ The tumor-specific proteins GFAP and S100 play crucial roles in diagnosing gliomas, and GFAP is recognized as the most extensively utilized astrocyte marker.^[99,100]^ Numerous studies suggest an association between the overexpression of CD34 and CD105 and higher WHO-grade gliomas, suggesting the possibility of discovering novel prognostic and diagnostic markers and therapeutic targets.^[101,102]^ Marina et al documented a case of malignant intrasellar-suprasellar glioma that originated from the pituitary stalk. The patient exhibited symptoms such as amenorrhea, headache, impaired vision, and hyperprolactinemia, and MRI revealed a thickened mass in the pituitary stalk. Surgery and radiotherapy alleviated the symptoms; however, complications such as DI and hypopituitarism emerged, necessitating long-term hormone replacement therapy.^[98]^ Hardian et al reported a patient initially diagnosed with astrocytoma that subsequently deteriorated and progressed to glioblastoma. The patient underwent multiple surgeries and chemotherapy, and the tumor gradually invaded the pituitary gland. The efficacy of the use of bevacizumab was questioned: it was suggested in the paper that the significant improvement in radiological response after treatment may have been only an appearance of tumor shrinkage. Although this study does not explicitly mention the involvement of the pituitary stalk, it may provide some insight into the actual effects of bevacizumab treatment.^[103]^

#### 3.2.3. Craniopharyngioma

Craniopharyngioma, a rare epithelial tumor primarily situated in the saddle/para saddle region, is widely considered by various authors to originate from the transformation of epithelial cells derived from the ectodermal remnants of Rathke’s bursa and craniopharyngioma.^[7,104]^ Although histologically classified as benign, it frequently infiltrates vital adjacent structures (hypothalamus, pituitary/pituitary stalk, optic crossings, etc) and frequently induces associated secondary diseases.^[104]^ Involvement of the pituitary stalk site in craniopharyngiosis is relatively uncommon. Dong et al compiled a summary of 45 cases of craniopharyngiomas in patients exhibiting thickened pituitary stalks.^[26]^ The genetic pathogenesis of craniopharyngiomas may be associated with BRAF V600E mutations and B-catenin mutations. A thorough investigation of this genetic mechanism opens up new possibilities for targeted therapy in craniopharyngiomas.^[104,105]^ The clinical presentation involves secondary signs/symptoms, including hypothalamic-pituitary dysfunction, CDI, increased intracranial pressure, and visual impairment. Treatment options primarily include surgery and radiotherapy, with surgery typically considered the primary choice.^[104]^ In the literature, Yoshikazu et al reported that when dealing with craniopharyngiomas originating from or metastasizing to the pituitary stalk, removing the tumor along with the pituitary stalk can effectively prevent regrowth within this region.^[106,107]^

#### 3.2.4. Others

Pituitary adenoma is a benign tumor that originates in the anterior pituitary gland and is among the most common primary intracranial tumors. While uncommon, there are instances where it may be solitary or arise in the pituitary stalk. Small adenomas are limited to the sella turcica, and as the tumor enlarges, the tumor can extend to impact regions such as the suprasellar and optic chiasmatic areas.^[7,108]^ Meningioma is a frequently encountered primary central nervous system tumor, and its clinical presentation varies based on the affected site.^[109]^ Involvement of the pituitary gland or pituitary stalk has been infrequently documented in the literature. On MRI, meningiomas affecting the pituitary stalk may exhibit thickening, and compression of the stalk could result in symptoms such as hypopituitarism and hyperprolactinemia. Following surgical resection, pituitary growth hormone levels normalize in the majority of patients, and exacerbation of hypopituitary symptoms is rarely observed.^[110]^ However, the deep localization of the tumor, the presence of numerous vascular and neural coverings, and the susceptibility of the pituitary stalk to involvement contribute to the complexity of surgical treatment for meningiomas.^[111]^ Several other uncommon primary tumors can cause thickening of the pituitary stalk. Central nervous system lymphomas and pituitary cell tumors were documented in the literature by Hána et al.^[112]^

Pituitary stalk metastases are prevalent in elderly patients with widespread malignant diseases. Regarding the type of metastasis, lung cancer is the most common primary cancer, followed by breast cancer and lymphoma.^[5,7,26]^ Other metastatic tumors that may lead to pituitary stalk invasion include leukemia, thyroid cancer, gastric cancer, and skin cancer. Typically, these metastases manifest as locally invasive lesions and exhibit a robust growth tendency. Common clinical indicators include symptoms such as DI and potentially hypopituitarism.^[7,10]^ On MRI, metastatic tumors typically exhibit thickening of the pituitary stalk.^[26]^

As of February 2021, Dong et al conducted a review of diverse tumors associated with the thickening of the pituitary stalk. These included one case of cavernous hemangioma, one case of choroid plexus carcinoma, one case of nerve sheath tumor, and one case of spindle cell carcinoma.^[26]^

### 3.3. Congenital

#### 3.3.1. Pituitary stalk interruption syndrome

Pituitary stalk interruption syndrome (PSIS) is a distinct developmental defect of the pituitary gland identified by MRI and characterized by a thin, interrupted, attenuated, or absent pituitary stalk, hypoplasia of the adenohypophysis, and an ectopic posterior pituitary.^[113]^ PSIS was initially proposed in 1987 by I. Fujisawa and T. Momoi et al.^[114]^ In a retrospective study involving 231 patients with hypopituitarism, Fernandez-Rodriguez et al reported that hormonal dysfunction was not significantly associated with congenital perinatal injuries or breech births. This implies that although perinatal injuries or breech delivery might have an impact on PSIS, they cannot be definitively identified as etiological factors.^[115–122]^ In their study of 6 patients with PSIS, Gardijan et al reported that half of the patients’ mothers had varying degrees of hormonal disorders. Therefore, it can be hypothesized that hormonal disorders in mothers may be related to PSIS to some extent, although this correlation needs further verification.^[123]^ It is widely acknowledged that the PSIS may be linked to genetic variants that are crucial to hypothalamic-pituitary development pathways. Genetic variants are primarily categorized as single-gene variants, double-gene variants, gene rearrangements, polygenic hypotheses, suspected environmental influences, or unknown types.^[113,116]^ Table 2 presents a synthesis of recent studies investigating genetic factors.^[124–138]^ These studies, conducted by prominent researchers, reveal that the majority of genetic variants are principally linked to midline development, pituitary function and development, hypogonadotropic hypogonadism, hypoplasia of the corpus callosum, and axonal migration. Additionally, recent literature has introduced new gene variants associated with PSIS. For instance, mutations in the KCNJ11 gene may correlate with cirrhosis and diabetes mellitus.^[124]^ Furthermore, mutations in the COL1A1 gene have been identified in individuals with osteogenesis imperfecta concurrent with pituitary stalk disruption syndrome.^[125]^ Notably, the CDON gene may be implicated in pituitary stalk disruption syndrome coupled with abducens nerve palsy.^[138]^

**Table 2:** The classification of genes associated with pituitary stalk interruption syndrome (PSIS).

Classifications	Gene
Midline development and/or pituitary development or function	BMP4,CDON, GLI2, GLI3, HESX1, KIAA0556, LHX4, LHX9, NKX2-1, PROP1, PTCH1, SHH, TBX19,TGIF1,PROKR2, WDR11,SOX3,NBPF9, GPR161, SIX3, FOXL2
Syndromic and non-syndromic forms of hypogonadotropic hypogonadism	CCDC141, CHD7, FANCA, FANCC, FANCD2, FANCE, FANCG, IL17RD, KISS1R, NSMF, PMM2, SEMA3E, WDR11, NR0B1
Syndromic forms of short stature	FGFR3, NBAS, PRMT7, RAF1, SLX4, SMARCA2, SOX11
Cerebellum atrophy and/or optic anomalies	DNMT1, NBAS
Axonal migration	ROBO1, SLIT2
Agenesis of the corpus callosum	ARID1B, CC2D2A, CEP120, CSPP1, DHCR7, INPP5E, VPS13B, ZNF423
Others	KCNJ11, COL1A1

The typical clinical presentation of PSIS involves a persistent deficiency in anterior pituitary hormones. Patients exhibit substantial hormone deficiencies at birth, which progressively worsen during childhood and often progress to total pituitary hypopituitarism with age.^[113]^ The primary hormones deficient in patients include GH, luteinizing hormone, FSH, ACTH, corticotropin-releasing hormone, and TSH. Growth hormone deficiency is the most prevalent hormone deficiency and is observed in almost all patients.^[118]^ Prolactin levels are significantly influenced by the degree of disruption of the dopaminergic pathway and may present as prolactin deficiency or hyperprolactinemia. However, CDI is relatively rare.^[113,129,139–141]^ In a study by Fernandez et al., 8.3% of patients with PSIS exhibited symptoms of polydipsia and polyuria, but further confirmation of DI is needed. Approximately 20% to 50% of patients may experience common congenital developmental anomalies, primarily affecting midline structures such as diaphragmatic agenesis, cleft lip and palate, and axial skeletal anomalies. The most prevalent monster is optic nerve hypoplasia, possibly linked to the migration of neural crest cells in the embryonic period.^[123]^ The clinical presentation of PSIS exhibits some heterogeneity, with certain patients initially manifesting specific symptoms such as epilepsy, mental deficits, and micropenis, aiding in its diagnosis.^[129]^ In a retrospective study involving 55 Chinese patients with PSIS, Guo et al observed distinct clinical characteristics in Chinese patients characterized by a greater degree of pituitary hypoplasia and a lower incidence of midline defect disease than in other reported cases.^[117]^

The diagnosis of pituitary stalk block syndrome (PSIS) relies on clinical manifestations of hormone deficiency and structural abnormalities in the pituitary stalk, as revealed by MRI.^[116]^ PSIS, characterized by complex etiology and pathogenesis, displays varied imaging features including anterior pituitary absence or hypoplasia, posterior pituitary absence, ectopic pituitary stalk, ectopic hypothalamic base, pituitary stalk interruption, and thinning.^[113,142,143]^

Early detection and treatment of hormone deficiency are critical to ensuring the quality of life and prognosis of patients with PSIS. Lifelong hormone replacement therapy is typically employed for patients diagnosed with PSIS due to the challenging nature of restoring damage to the pituitary stalk through surgery. Following a comprehensive evaluation of anterior pituitary hormone levels, replacement therapy should be tailored to the specific hormone deficiency. The sequence of replacement therapy may involve Adrenocortical hormone-thyroxine-growth hormone-sex hormones, and continuous long-term follow-up and review are necessary to evaluate the effectiveness of hormone replacement therapy.^[121,144,145]^

#### 3.3.2. Pituitary hypoplasia

Pituitary hypoplasia primarily results from incomplete development of brain tissue or insufficient nerve cells. This may be linked to perinatal injuries, including fetal cerebral hypoplasia, obstructed labor, birth injury, birth asphyxia, intracranial hemorrhage in newborns, febrile convulsions, encephalitis, meningitis, and traumatic brain injury. Genetic factors may also contribute to its occurrence. Previous literature summaries have frequently linked ROBO1 gene variants to pituitary stalk blockage syndrome. For example, a study conducted by Marcello Scala et al reported anterior pituitary hypoplasia and pituitary stalk duplication in a girl with a deletion of the ROBO1 gene (whose father exhibited a similar phenotype), thus expanding the spectrum of ROBO1 gene phenotypes.^[146]^ A study by Pierluigi et al suggested that mutations in the cAMP-binding protein-BP gene CREBBP may be linked to pituitary dysplasia.^[147]^ Additionally, Keigo et al discovered that the knockout of mitochondrial ubiquitin ligase may induce anterior pituitary dysplasia, confirming the pivotal role of mitochondrial ubiquitin ligase in the development of the anterior pituitary.^[148]^ Clinical manifestations of pituitary hypoplasia include dwarfism, growth hormone deficiency, hypothyroidism in children, delayed puberty due to sex hormone deficiency, and a childish appearance.^[5,10]^ MRI can reveal various abnormalities related to the development of the pituitary stalk, including pituitary stalk agenesis, thickening, shortening, ectopic posterior pituitary, and anterior pituitary dysplasia.^[5,10,11,40,148]^ Surprisingly, a cross-sectional study conducted by Himanshu Sharma et al revealed that pituitary hypoplasia is the most reliable predictor of various saddle zones and extra-saddle congenital idiopathic growth hormone deficiency as well as multiple pituitary hormone deficiencies.^[149]^ The primary treatment for this disorder involves hormone replacement therapy, and the earlier the treatment is initiated, the more favorable the outcomes tend to be.^[10]^

#### 3.3.3. Pituitary stalk duplication

The exact etiology of double pituitary stalk malformations remains inconclusive, with the most widely accepted theory proposing that splitting of the anterior end of the notochord results in replication of the neural ectodermal adherent zone, thereby giving rise to 2 pituitary glands.^[150]^ The presence of this double pituitary stalk lesion has been reported in multiple publications and is linked to a relatively high infant mortality rate. Facial midline abnormalities frequently characterize such cases, and reports indicate a potential association between these double pituitary stalk malformations and deletion of the ROBO1 gene.^[5,10,40,146,150–158]^ In a study conducted by Mohamed et al, magnetic resonance findings revealed double pituitary stalk lesions in a patient with morning glory optic disk anomaly, indicating the potential involvement of pituitary stalk replication in the pathogenesis of morning glory optic disk anomaly.^[156]^ The main diagnostic tool for this condition is MRI, which reveals 2 pituitary glands, each with a stalk. MRI and CT scans complement each other in assessing these complex lesions, with CT scans providing superior visualization of skull base defects and areas of calcification. Moreover, coronal MR images anatomically reveal saddle zones and tiny fat foci. Therefore, a combination of both imaging methods may be necessary in some instances.^[10,153]^ Currently, no effective treatment for pituitary stalk duplication has been identified. Nevertheless, Kandpal et al administered pituitary hormone replacement therapy and bromocriptine to one of their patients, and receiving favorable feedback on the therapy.^[153]^

#### 3.3.4. Rathke cleft cyst

Rathke’s cyst (RCC) is a benign lesion situated in the saddle region and/or suprasellar area that originates from the embryonic remnant of Rathke’s bursa; this type of lesion is relatively rare and particularly underrepresented in the literature for pituitary stalk lesions.^[159,160]^ In their study on the etiology of pituitary stalk lesions, Mirjana Doknic and colleagues included only 2 out of 53 patients with Rathke’s cysts.^[42]^ Raluca Trifanescu and colleagues suggested that the embryonic residual Rathke’s bursa extends intracranially to form the craniopharyngeal canal. Failure of the canal to occlude results in the development of cysts between the distal and neural portions of the canal, leading to the formation of Rathke’s cysts.^[161]^ Rathke’s cysts typically lack typical clinical symptoms but may manifest as hypopituitarism, visual field defects, episodic headache, DI, hyperprolactinemia, or compression symptoms.^[7,159,160,162]^ MRI and CT are the primary radiological methods employed for lesion examination. CT typically reveals low-density cystic areas but may also indicate homogeneous lesions or mild hyperdensities concerning the brain parenchyma. MRIs exhibit variability and complexity, with signal intensity correlated with the protein content, predominantly reflecting a low signal intensity.^[7,72,159,161,162]^ When it accumulates in the pituitary stalk, it can manifest as a non-enhancing cystic mass restricted to the pituitary stalk.^[42,161]^ Nevertheless, a study by Daniela Dadej et al^[68]^ revealed that among 102 patients with RCC, 11 exhibited pituitary compression, and 6 displayed specific manifestations of pituitary stalk subluxation.^[160]^ Michelle L Brinkmeier et al identified the transcription factor ISL1 as a potential pathological predictive marker for diagnosing Rathke’s cysts.^[163]^ Treatment options include transsphenoidal surgical drainage, excision, or observation, and surgery is generally not recommended for asymptomatic patients.^[159,161,162]^ TSS is a commonly performed procedure; however, DI may occur as a complication in the days following surgery. Yasuhiko et al discovered that the delayed onset of DI after TSS in patients with RCC may be linked to the spread of inflammation to the pituitary stalk (the funiculus) following RCC resection.^[164]^

#### 3.3.5. Septooptic dysplasia

Septo-optic dysplasia, also known as de Morsier syndrome, is a relatively rare congenital disorder characterized by the classic triad of cerebral pellucid septal defects, optic nerve hypoplasia, and hypopituitarism.^[5,42,165–169]^ Previous studies have suggested an association between SOD and HESX1, SOX2, and SOX3 variants.^[166]^ In a genetic study, Linda M Reis et al identified novel genetic variants in SOX2, SHH, and ARID1A in a family cohort diagnosed with SOD.^[167]^ In their groundbreaking report, Fernández-Marmiesse et al revealed for the first time that variants in the FLNA gene were associated with SOD.^[169]^ SOD can cause various other conditions, such as gestational injuries (e.g., viral infections, gestational diabetes) and vascular disruption.^[5,170,171]^ SOD can accumulate in the visual system, nervous system, and hypothalamic-pituitary endocrine system, etc.^[168,169,172–174]^ The primary clinical manifestation of SOD is hormone deficiency, which can be caused by growth hormone, ACTH, thyroid stimulating hormone, or antidiuretic hormone.^[5,165]^ MRI of SOD may reveal anesis of the septum and corpus callosum, along with issues related to the development of the pituitary stalk and posterior pituitary lobe.^[175]^ Previous reports have shown that SOD involving the pituitary stalk can result in symptoms such as anterior pituitary hypoplasia, ectopic posterior pituitary, pituitary stalk thinning or absence, and an empty sella turcica.^[5,165]^ In a recent study, Ouazzani et al reported a case in which a boy with SOD had an abnormally thin pituitary stalk on MRI, which is consistent with the findings of previous studies.^[175]^ There is no established standard treatment for this disease, and the primary approach involves symptomatic treatments, including addressing visual and intellectual impairments and hormone replacement therapy.^[173,175]^ Due to its rarity and significant association with hypopituitarism, early diagnosis and treatment of patients with SOD are crucial, emphasizing a multidisciplinary approach.^[165,176]^

## 4. Conclusion

In conclusion, the diversity of diseases leading to pituitary stalk lesions, coupled with their similar symptoms, complicates their identification and diagnosis in clinical practice. The ongoing use and advancement of imaging technologies, such as MRI and PET-CT, have established a foundation for clinical diagnosis and therapeutic decision-making. This article extensively discusses the etiological classification of various diseases causing pituitary stalk lesions, encompassing the etiology, pathological features, clinical manifestations, and diagnostic and therapeutic aspects of these diseases. Future advancements in high-throughput biotechnologies, including medical imaging, genomics, proteomics, and metabolomics, are expected to enhance the diagnostic accuracy of pituitary stalk lesions.

## Author contributions

**Funding acquisition:** Zonglan Chen.

**Writing – original draft:** Zaidong Zhang, Jinlin Wang.

**Writing – review & editing:** Yaru Shi, Yahui Zhao, Yanli Hu, Wentao Wang, Zonglan Chen.
